# Beyond the norm: A rare case of cardiac and extracardiac metastatic urothelial carcinoma

**DOI:** 10.1002/ccr3.8025

**Published:** 2023-10-09

**Authors:** Ahmed Hassaan Qavi, Tehniat Fakhar, Mahnoor Khalid, Husam El Sharu, Soban Ahmad, Rony Shammas, Constantin Bogdan Marcu

**Affiliations:** ^1^ Department of Cardiovascular Sciences East Carolina University and Brody School of Medicine Greenville North Carolina USA; ^2^ Department of Medicine Shifa College of Medicine Islamabad Pakistan; ^3^ Department of Medicine Foundation University Islamabad Pakistan; ^4^ Department of Medicine East Carolina University and Brody School of Medicine Greenville North Carolina USA; ^5^ Division of Cardiovascular Medicine University of Nebraska Medical Center Omaha Nebraska USA

**Keywords:** cardiac metastasis, cardiac MRI, echocardiography, extracardiac metastasis, urothelial carcinoma

## Abstract

Screening echocardiography aids in identifying cardiac emboli causes and asymptomatic cardiac metastases in high‐grade neoplasms. Conversely, cardiac MRI provides advanced tissue characterization and broader extracardiac assessment.

## Graphical abstract

Apical four chamber view of transthoracic echocardiogram showing an irregular left ventricular mass attached to the apex, suspicious for a malignant tumor.

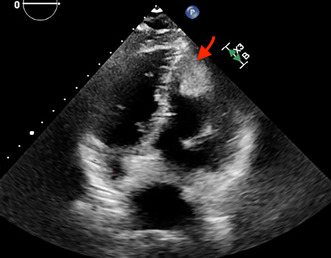

## 1 INTRODUCTION

Cardiac metastasis from urothelial cancer is a rare occurrence. In this report, we describe the case of a 63‐year‐old male with recently diagnosed metastatic urothelial cancer who presented with weakness in his right upper extremity. Subsequent brain imaging revealed numerous acute infarctions in multiple vascular territories. Further workup with multimodality cardiac imaging unveiled a left ventricular (LV) mass extending into the ventricular cavity.

## 2 CASE PRESENTATION

**FIGURE 1:**
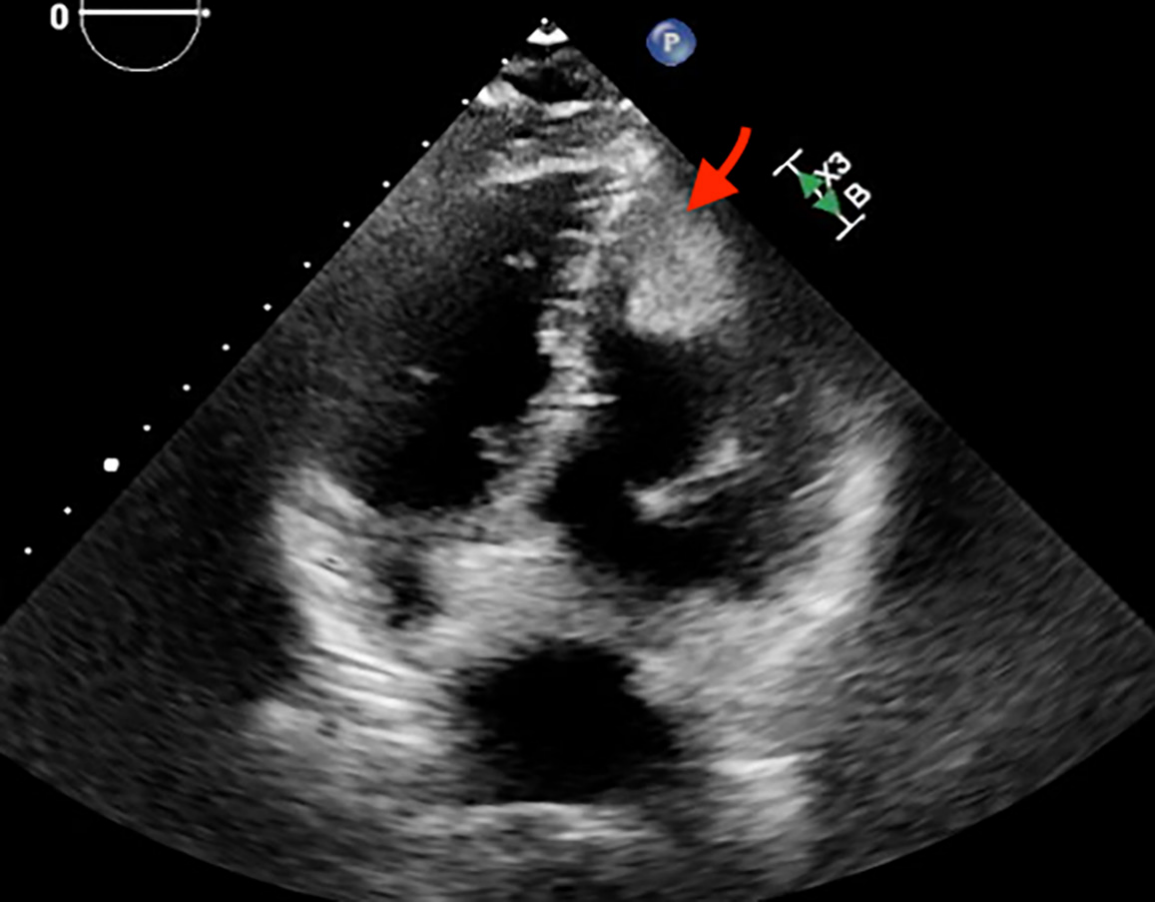
Transthoracic echocardiogram four chamber view demonstrating an irregular distal left ventricular mass (arrow).

**FIGURE 2:**
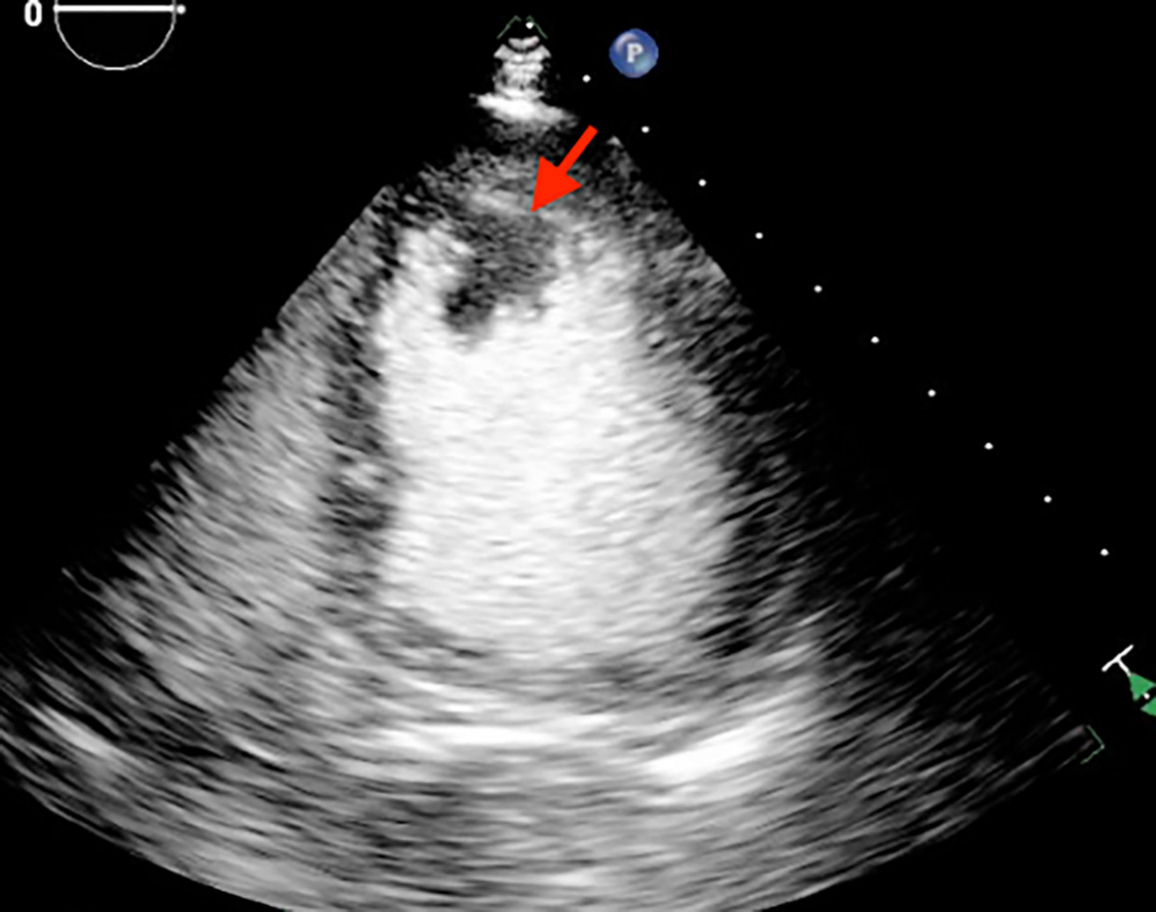
Transthoracic echocardiogram two chamber view with contrast demonstrating an irregular distal left ventricular mass (arrow).

**FIGURE 3:**
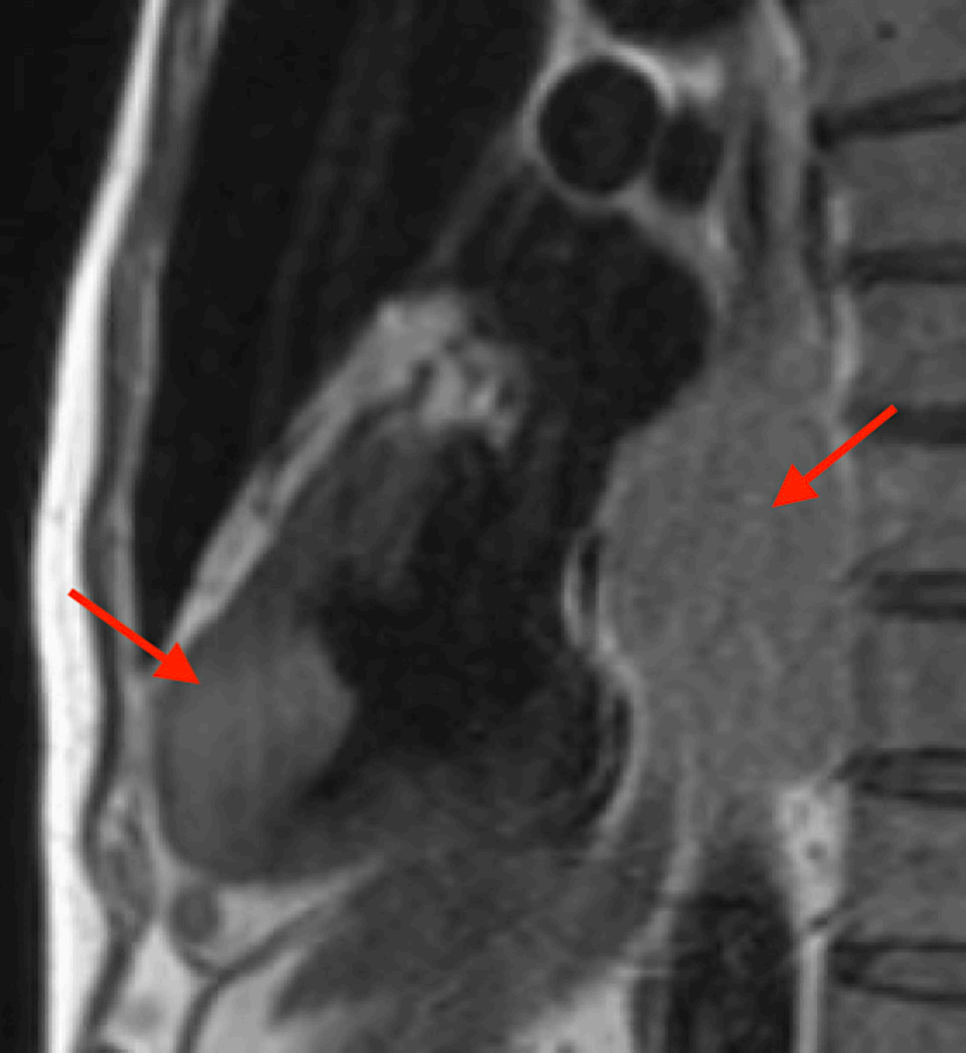
Cardiac MRI T2w TSE (turbo spin echo) pre Gd‐contrast, left ventricle apical and posterior to the LA masses with intermediate signal intensity (arrows).

**FIGURE 4:**
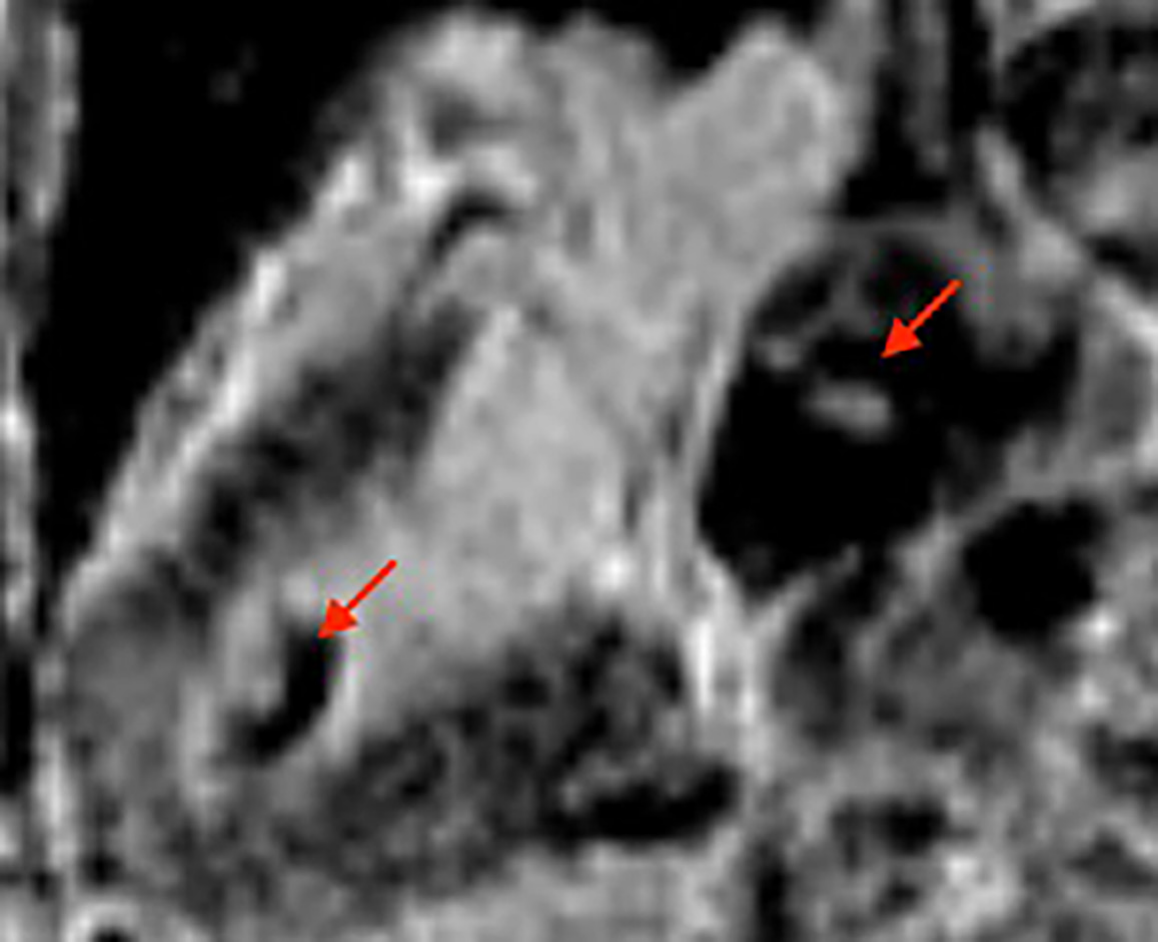
Cardiac MRI post Gd‐contrast IR (inversion recovery) LGE (late gadolinium enhanced) sequences showing peripheral enhancement with necrosis in the center of both masses (arrows). The masses have similar signal characteristics on CMR consistent with their common origin.

A 63‐year‐old male came to the emergency department with sudden‐onset weakness of his right arm. He had a significant medical history of high‐grade urothelial carcinoma and had undergone radical cystoprostatectomy with ileal conduit diversion 3 months prior to his current presentation. He had undergone contrast enhanced cardiac tomography of chest and abdomen for hypercalcemia workup prior to his prostate surgery and it did not demonstrate any evidence of cardiac metastasis. Laboratory testing at admission showed hemoglobin 10.1 g/dL (normal: 14–18 g/dL), sodium 129 mEq/L (normal: 135 and 145 mEq/L), and serum calcium 12.7 mg/dL (normal: 8.6–10.3 mg/dL). Electrocardiogram (ECG) showed sinus tachycardia with evidence of LV hypertrophy based on voltage criteria. Troponin levels peaked at 0.46 ng/mL (normal: <0.03 ng). Subsequent magnetic resonance imaging (MRI) of the brain exhibited multiple areas of acute infarction in both the cerebral and cerebellar hemispheres, suggesting a potential embolic cause. Considering the MRI findings and elevated troponin levels, a transthoracic echocardiogram (TTE) was performed, revealing a large sessile mass located in the distal part of the left ventricle (LV) without any abnormalities in wall motion and with a normal overall LV ejection fraction (LVEF) (Figures 1 and 2, Video S1). A CMR was performed for tissue characterization and showed an irregular mass in the LV myocardium and cavity and a new mediastinal mass posterior to but not invading the left atrium (Figures 3 and 4). Also noted were multiple round lung lesions. The cardiac mass demonstrated myocardial invasion without pericardial involvement. It had high signal intensity on native T2‐weighted sequences, intermediate signal intensity on native T1‐weighted sequences while on both early and late post gadolinium‐contrast sequences it showed peripheral enhancement with central low signal intensity, suggesting increased peripheral vascularity with central necrosis. The cardiac and extracardiac masses had similar pre‐ and post‐contrast signal characteristics, suggesting a common origin (metastatic disease). Due to the presence of metastatic disease and the poor overall prognosis, the patient received palliative treatment and died peacefully.

## 3 DISCUSSION

Our case highlights the rare occurrence of cardiac metastasis associated with advanced‐stage urothelial carcinoma. While primary cardiac tumors are uncommon, with a prevalence of up to 0.1%, metastatic cardiac tumors have been identified in up to 9% of oncologic patients.^1^ Urothelial cardiac metastasis, although exceedingly rare, has been reported in a limited number of cases, with multiple cardiac metastatic lesions reported in only three cases to date.^2^, ^3^, ^4^

Intracavitary cardiac metastasis with urothelial carcinoma has not been reported previously. It has been typically reported with metastatic renal or hepatic carcinoma and can lead to embolic phenomenon, ventricular outflow obstruction, and cardiogenic shock.^1^ Additionally, myocardial involvement can lead to conduction abnormalities, systolic and diastolic dysfunction, myocardial infarction and rupture, leading to cardiac tamponade and death.^2^

Although it can be challenging to identify the cancerous origin of a cardiac lesion without biopsy, factors such as a known primary malignancy, metastatic spread, and noninvasive tissue characterization through cardiac imaging can aid in determining the etiology of cardiac mass. Advances in multimodality imaging techniques have proven to be invaluable in differentiating metastatic cardiac disease from other conditions. Echocardiography can detect malignant pericardial effusion, local myocardial dysfunction, and myocardial masses. Additionally, contrast‐enhanced cardiac magnetic resonance (CMR) imaging is particularly valuable in accurately identifying and localizing cardiac metastatic lesions, as well as differentiating them from intracavitary thrombi, infectious lesions, and other benign conditions.^5^ Metastatic lesions mostly appear as irregular, enhancing masses with the presence of surrounding tissue infiltration whereas benign cardiac lesions such as fibromas or myxomas have well‐defined borders and homogenous appearance with the lack of tissue invasion on CMR. Cardiac thrombi on the other hand have low signal intensity on both T1‐weighted and T2‐weighted images with no contrast enhancement. Finally, infectious lesions such as endocarditis or myocarditis present as areas of inflammation which on CMR appear as areas of increased signal intensity on T2‐weighted images hand.^5^, ^6^ F18‐fluorodeoxyglucose (FDG) positron emission tomography‐computed tomography (PET‐CT), while useful in some cases, requires a more cautious interpretation as myocardial uptake can vary physiologically, and benign lesions can have increased PDG‐uptake mimicking malignancy.^6^

Treatment approaches for cardiac metastasis depend on the clinical presentation, with pericardiocentesis and radiofrequency ablation used to manage cardiac complications like tamponade and arrhythmias.^7^ Overall, urothelial cancer with cardiac metastasis is a rare condition associated with a poor prognosis. Therefore, the main goal of management is to relieve obstruction and other cardiac complications through short‐term radiotherapy or chemotherapy for palliative purposes. Surgical resection can be considered in isolated intracavitary cardiac lesions in exceptional cases with otherwise favorable prognoses.^8^

In conclusion, cardiac metastasis in patients with urothelial carcinoma presenting with stroke‐like symptoms should be strongly considered. Multimodality cardiac imaging can assist in definitive diagnosis. Screening TTE serves as a valuable tool to diagnose asymptomatic cardiac metastases in cases of high‐grade neoplasms. In contrast, cardiac MRI offers providing enhanced tissue characterization and a comprehensive view of any involvement beyond the heart. Palliative management should be considered to alleviate symptoms and improve quality of life.

## AUTHOR CONTRIBUTIONS

**Ahmed Hassaan Qavi:** Conceptualization; investigation; supervision; writing – original draft; writing – review and editing. **Tehniat Fakhar:** Investigation; writing – original draft. **Mahnoor Khalid:** Investigation; methodology; writing – original draft. **Husam El Sharu:** Writing – original draft. **Soban Ahmad:** Investigation; methodology; writing – review and editing. **Rony Shammas:** Conceptualization; supervision. **Constantin Bogdan Marcu:** Supervision; writing – review and editing.

## CONSENT

Written informed consent was obtained from the patient to publish this report in accordance with the journal's patient consent policy.

## Supporting information


Video S1.

## Data Availability

Data sharing is not applicable to this article as no new data were created or analyzed in this study.
